# *Entamoeba histolytica* Trophozoites Induce a Rapid Non-classical NETosis Mechanism Independent of NOX2-Derived Reactive Oxygen Species and PAD4 Activity

**DOI:** 10.3389/fcimb.2018.00184

**Published:** 2018-06-05

**Authors:** César Díaz-Godínez, Zayda Fonseca, Mario Néquiz, Juan P. Laclette, Carlos Rosales, Julio C. Carrero

**Affiliations:** ^1^Laboratory of Immunology, Department of Immunology, Instituto de Investigaciones Biomédicas, Universidad Nacional Autónoma de México, Mexico City, Mexico; ^2^Laboratory of Immunopathology, Department of Experimental Medicine, Hospital General de México, Facultad de Medicina, Universidad Nacional Autónoma de México, Mexico City, Mexico

**Keywords:** *Entamoeba Histolytica*, neutrophils, NETosis, NETs, ROS, NOX2, PAD4

## Abstract

Neutrophil extracellular traps (NETs) are DNA fibers decorated with histones and antimicrobial proteins from cytoplasmic granules released into the extracellular space in a process denominated NETosis. The molecular pathways involved in NETosis have not been completely understood. Classical NETosis mechanisms involve the neutrophil elastase (NE) translocation to nucleus due to the generation of reactive oxygen species (ROS) by NADPH oxidase (NOX2) or the peptidyl arginine deiminase 4 (PAD4) activation in response to an increase in extracellular calcium influx; both mechanisms result in DNA decondensation. Previously, we reported that trophozoites and lipopeptidophosphoglycan from *Entamoeba histolytica* trigger NET release in human neutrophils. Here, we demonstrated in a quantitative manner that NETs were rapidly form upon treatment with amoebic trophozoites and involved both nuclear and mitochondrial DNA (mtDNA). NETs formation depended on amoeba viability as heat-inactivated or paraformaldehyde-fixed amoebas were not able to induce NETs. Interestingly, ROS were not detected in neutrophils during their interaction with amoebas, which could explain why NOX2 inhibition using apocynin did not affect this NETosis. Surprisingly, whereas calcium chelation reduced NET release induced by amoebas, PAD4 inhibition by GSK484 failed to block DNA extrusion but, as expected, abolished NETosis induced by the calcium ionophore A23187. Additionally, NE translocation to the nucleus and serine-protease activity were necessary for NET release caused by amoeba. These data support the idea that *E. histolytica* trophozoites trigger NETosis by a rapid non-classical mechanism and that different mechanisms of NETs release exist depending on the stimuli used.

## Introduction

Neutrophils are the most abundant leucocytes in the mammal peripheral blood. Neutrophils have different strategies to combat pathogens including phagocytosis, degranulation and production of neutrophil extracellular traps (NETs). NETs are DNA fibers decorated with histones and antimicrobial proteins from cytoplasmic granules (Papayannopoulos and Zychlinsky, 2009) that are combined in the cytosol and released into the extracellular space in a process denominated NETosis (Steinberg and Grinstein, 2007). NETosis has been described as a cellular death process different from apoptosis and necrosis as it is independent of caspase pathway and there is no phosphatidylserine exposition on cell surface. In addition, NETosis is culminated with DNA extrusion (Fuchs et al., 2007).

The molecular pathways involved in NETosis has not been completely understood. During phorbol miristate acetate (PMA) stimulation, NADPH oxidase (NOX2) assemble take place to produce reactive oxygen species (ROS) (Fuchs et al., 2007). ROS promote the translocation of neutrophil elastase (NE) from the cytoplasmic granules to the nucleus where this enzyme cleaves histones to promote DNA decondensation (Papayannopoulos et al., 2010). Nevertheless, calcium ionophores ionomycin and A23187 appear to induce NETosis independently of NOX2-derived ROS; however, they depend on peptidyl arginine deiminase 4 (PAD4) activity to promote DNA decondensation by changing positive charged arginine residues to none charged citrulline residues in histones (Wang et al., 2009). This mechanism occurs due to an increase in extracellular calcium influx that activates PAD4 (which has four sites for calcium binding), and theoretically any molecule with the capacity to form pores in the cytoplasmic membrane of neutrophils (e.g., bacterial toxins or complement) are able to induce NETosis through this pathway (Konig and Andrade, 2016). Additionally, mitochondrial DNA (mtDNA) NETosis (Yousefi et al., 2009) has been reported in neutrophils which were primed with granulocyte/macrophage-colony stimulatory factor (GM-CSF) and stimulated with lipopolysaccharide (LPS) or complement fraction C5a; this form of NETs release was named vital NETosis because only mtDNA is extruded without compromise cell viability of neutrophils, in contrast with the lethal nuclear NETosis.

*Entamoeba histolytica* is an intestinal parasite with high prevalence in developing countries (Tellevik et al., 2015; Ghenghesh et al., 2016). Neutrophils have been implicated in defense against this parasite playing a crucial protective role (Asgharpour et al., 2005); nevertheless, involvement of neutrophils and other leukocytes in tissue damage has also been reported (Olivos-García et al., 2007). Oxidative (ROS production) and non-oxidative mechanisms are proposed to be used by neutrophils to kill amoeba (Ghosh et al., 2010; Pacheco-Yépez et al., 2011; Campos-Rodríguez et al., 2016). Mechanisms triggering neutrophils by amoebas are unknown but they could involve innate immune receptors such as TLRs. In this sense, human monocytes TLR2 and TLR4 recognition of lipopeptidophosphoglycan (LPPG) present on cell surface of amoebic trophozoites results in IL-6 and TNF-α release, suggesting that at least amoebic LPPG can activate immune cells trough PRRs (Maldonado-Bernal et al., 2005). Previously, *in vitro* NETs production in response to *E. histolytica* trophozoites or the LPPG from this parasite was demonstrated by our group (Ávila et al., 2016). Interestingly, non-viable amoebas failed to induce NETosis and trophozoites treated with PMA-derived NETs did not decreased neither their viability nor the capacity to develop amoebic liver abscess in a hamster model (Ávila et al., 2016). The mechanism of NET induction by *E. histolytica* is still unknown and its characterization could contribute to our understanding of the still controversial role of neutrophils in amoebiasis.

Here, we demonstrated that *E. histolytica* trophozoites rapidly induced NETosis by a mechanism independent of NOX2-derived ROS and PAD4 activity; however, this mechanism was dependent on the presence of extracellular calcium and serine-protease activity. These data support the notion of the existence of different NETosis processes that are triggered depending on the stimuli used, the study of which may add to the understanding of the role of these innate immunity mechanisms in parasitic infections.

## Materials and methods

### *Entamoeba histolytica* trophozoites

*Entamoeba histolytica* trophozoites (strain HM1:IMSS) were cultured axenically at 37°C in TYIS-33 medium supplemented with 15% heat-inactivated adult bovine serum and Diamond vitamin tween solution (Sigma). The cultures were incubated for 72 h and trophozoites were harvested by chilling on ice for 5 min and centrifugation at 1,500 rpm for 5 min at 4°C. The pelleted amoebas were resuspended in PBS pH 7.4 and counted. For some experiments, trophozoites were washed with PBS and formaldehyde-fixed or heat-inactivated at 56°C during 10 and 30 min, respectively.

### Neutrophil isolation

Neutrophils were isolated from peripheral blood of healthy volunteers using Ficoll-Paque gradient (GE Healthcare) and hypertonic shock to lyse erythrocytes, as previously described (García-García et al., 2013). Cells were resuspended in RPMI medium supplemented with 5% fetal bovine serum and kept until use at 4°C. This study was carried out in accordance with the recommendations and approval of the Ethical Committee for Studies on Humans of the Instituto de Investigaciones Biomédicas, UNAM. All subjects gave written informed consent in accordance with the Declaration of Helsinki.

### NET quantification assay

Neutrophils (3 × 10^5^ cells) were stimulated by co-culturing with viable or fixed or heat-inactivated non-viable *E. histolytica* trophozoites (1.5 × 10^4^ cells) in 500 μL of RPMI during 4 h at 37°C under 5% CO_2_ atmosphere; 20 nM PMA was used as positive control of NETosis instead of trophozoites. In selected experiments 10 μM of the calcium ionophore A23187 was used as NETosis inducer. After stimulation, supernatants were collected by centrifugation at 4,000 rpm for 2 min to eliminate cells and 50 μL of the supernatants were added to the wells of a 96 well-plate containing 50 μL of PBS pH 7.4 with 500 nM SYTOX Green (Thermo-Fisher). Fluorescence was read from the bottom of wells in a spectrofluorometer using 485 nm excitation and 528 nm emission filters.

To obtain fluorescence values during the first 1 h of stimulation, neutrophils (1 × 10^5^) were co-cultured with viable *E. histolytica* trophozoites (5 × 10^3^) in 100 μL RPMI added with 500 nM SYTOX Green. Fluorescence values were measured each 5 min during 1 h with the 485 nm excitation and 528 nm emission filters as above.

In parallel experiments, neutrophils were pre-incubated before each stimulation with either DMSO-dissolved inhibitors apocynin (200 μM), PMSF (1 mM), or GSK484 (10 μM) at 4°C during 30 min. In the experiments with EGTA (20 mM), it was added 20 min before stimulation.

### NET visualization

#### Single DNA staining

NETs staining was performed by co-culturing 2 × 10^5^ neutrophils with 1 × 10^4^ *E. histolytica* trophozoites (20:1 ratio) in 200 μL RPMI medium using Chamber Slide™ System (Lab-Tek) for the respective time-points at 37°C under 5% CO_2_ atmosphere. After indicated time-periods, cultures were fixed with 4% formaldehyde for 10 min, washed three times with PBS and stained with 5 μg/mL DAPI. The coverslips were mounted with Fluoroshield (Sigma) before observation in a fluorescence microscope (Olympus BX51).

#### Immunofluorescence

NETs were induced with *E. histolytica* trophozoites on the Chamber Slide™ System as described above, and the cells were fixed with 4% paraformaldehyde for 10 min. The fixed cells were then permeabilized by adding 0.2% Triton X-100 in PBS for 5 min. Detergent was washed out three times with cold PBS and unspecific protein binding was blocked with a solution of 1% BSA, 0.3 M glycine, 0.1% Tween 20 in PBS for 30 min at 37°C. The samples were incubated with primary anti-NE (Santa Cruz Biotechnology), anti-myeloperoxidase (Abcam), anti-histone H4 (Abcam), or anti-citrulline (Abcam) antibodies diluted 1:100 in 1% BSA, 0.1%Tween 20 in PBS during 1 h at room temperature. The cells were gently washed with cold PBS and incubated with secondary anti-mouse IgG-FITC (Sigma) or anti-rabit IgG-TRITC (Zymax) antibodies diluted 1:50 in the same solution that primary antibody for 1 h at room temperature in darkness. The cells were then washed with PBS and stained with 5 μg/mL DAPI. The coverslips were mounted with Fluoroshield (Sigma) before observation in a fluorescence microscope (Olympus BX51).

#### Scanning electron microscopy

For scanning electron microscopy, neutrophils (3 × 10^5^) were co-cultured with *E. histolytica* trophozoites (1.5 × 10^4^) in RPMI medium on cover glass previously coated with poly-lysine. After 4 h of incubation, samples were fixed with 2.5% glutaraldehyde, washed with PBS and dehydrated by increasing concentrations of ethanol. Samples were air dried and coated with gold using Fine Coat Ion Sputter before analysis in an electron microscope Jeol JSM-7600F.

### Apoptosis and necrosis detection

#### DNA fragmentation assay

DNA integrity assay was performed by incubating neutrophils under different conditions. Neutrophils were stimulated with 20 nM PMA or 10 μM A23187 or *E. histolytica* trophozoites at 37°C for 1 h to induce NETosis. Necrosis, and in a lesser extent apoptosis, were induced by culturing neutrophils at 56°C for 1 h. The samples were centrifuged at 7,000 rpm during 5 min, the pellets were resuspended in 20 μL of lysis buffer (2 mM EDTA, 100 mM Tris-HCl, 0.8% SDS, pH 8.0) and 10 μL of 20 mg/mL proteinase K was added. Samples were incubated during 1.5 h at 56°C and mixed with DNA loading buffer. Finally, the samples were run in 1.5% agarose gel and stained with ethidium bromide for visualization.

#### Annexin V assay

Phosphatidylserine exposure was tested by FITC Annexin V Apoptosis Detection Kit I (Beckton Dickinson). In brief, necrotic cells (and in a lesser extent apoptotic cells) obtained after heat-treatment or NETotic cells obtained with PMA, A23187 or amoebas, as described above, were washed with PBS and then resuspended in binding buffer at a concentration of 1 × 10^7^ cells/mL. A total of 100 μL from the cell suspension were transferred to a new tube, and added with 5 μL of FITC annexin V. After gently vortexing, samples were incubated in darkness for 15 min at room temperature and observed by fluorescence microscopy. A total of 300 cells were counted to determine the percentage of apoptotic cells in the samples.

### ROS measurement

ROS generation was determined by flow cytometry using 2′,7′-dichlorodihydrofluorescein (H_2_DCFDA). In brief, 1 × 10^6^ neutrophils were pre-incubated with 10 μM H_2_DCFDA (Sigma) for 30 min at 37°C and washed three times with PBS. Cells were resuspended in 1 mL of PBS, stimulated with 5 × 10^4^ *E. histolytica* trophozoites or 20 nM PMA (positive control for ROS production) and incubated at 37°C. Cultures were fixed with 4% formaldehyde at 0 or 1 h. Finally, the 2′7′-dichlorofluorescein (DCF) fluorescence was measured using a FACScalibur cytometer. At least 1 × 10^4^ gated events of each sample were analyzed. Not stained and stained but not-stimulated cells were used as autofluorescence and negative controls, respectively.

### Amplification of nuclear and mitochondrial genes from NETs

#### Obtaining of NET solution

To obtain NETs solution, neutrophils (1 × 10^6^) were co-cultured with *E. histolytica* trophozoites (5 × 10^5^) in serum-free RPMI for 15 min. Supernatants were collected after centrifugation at 4,000 rpm for 2 min three times to eliminate residual cells. DNA concentration was measured with NanoDrop 2000.

#### Neutrophil DNA isolation

Neutrophils (4 × 10^6^) were lysed in 400 μl lysis buffer containing 1% SDS, 50 mM Tris–HCl at pH 8, 100 mM EDTA and 200 μg/ml proteinase K at 56 °C for 2 h. After addition of 125 μl of 5M NaCl solution, DNA was precipitated adding ice-cold isopropanol, re-suspended in 100 μL nuclease-free water, and extracted with phenol-chloroform-isoamyl alcohol (25:24:1). DNA was precipitated from aqueous fraction adding 0.25 M sodium acetate (pH 5.2) and absolute ethanol. After a final wash with ice-cold 70% ethanol, DNA was resuspended in water (Cotter and Muruve, 2006) and the concentration determined in NanoDrop 2000 (Thermo-Fisher).

#### Trophozoite DNA isolation

DNA from *E. histolytica* trophozoites was obtained as described elsewhere (Bhattacharya et al., 1988). Trophozoites from 72 h cultures were harvested by centrifugation and resuspended in 5 mL of NET Buffer containing 100 mM NaCI, 10 mM EDTA, 10 mM Tris, pH 8, and lysed with 0.2% SDS. DNA from suspension was extracted with phenol–chloroform (25:24) and precipitated with absolute ethanol. The precipitated DNA was dissolved in TNE Buffer containing 10 mM NaC1, 1 mM EDTA, 10 mM Tris, pH 8, treated with proteinase K at 37°C for 45 min, and extracted again with phenol-chloroform. After ethanol precipitation, the DNA was dissolved in TNE Buffer and its concentration was measured in NanoDrop 2000.

#### PCR conditions

PCR was perform using HotStarTaq® Plus Master Mix Kit (Qiagen) according to the manufacturer instruction. The primers used were as previously designed by Yousefi et al. (2009). For (I) amplification of mitochondrial genes: ATP synthase subunit 6 (*atp6*) (5′-ATACACAACACTAAAGGACGAACCT-3′ and 5′-GAGGCTTACTAGAAGTGTGAAAACG-3′), cytochrome oxidase c subunit 1 (*co1*) (5′-GGAGTCCTAGGCACAGCTCTAA-3′ and 5′-GGAGGGTAGACTGTTCAACCTG-3′), NADH dehydrogenase subunit 1 (*nd1*) (5′-GCATTCCTAATGCTTACCGAAC-3′ and 5′-AAGGGTGGAGAGGTTAAAGGAG-3′), cytochrome oxidase b (*cyb*) (5′-CTAGCAGCACTCCACCTCCTAT-3′ and 5′-GTTGTCCTCCGATTCAGGTTAG-3′); (II) amplification of nuclear genes: glyceraldehyde phosphate dehydrogenase (*gapdh*) (5′-CCCCTTCATTGACCTCAACTAC-3′ and 5′-GAGTCCTTCCACGATACCAAAG-3′), β-actine (*actb*) (5′-ATCTGGCACCACACCTTCTACAATGAGCTGCG-3′ and 5′-CGTCATACTCCTGCTTGCTGATCCACATCTGC-3′), and FAS receptor (*fas*) (5′-TCACCACTATTGCTGGAGTCAT-3′ and 5′-TAAACATCCTTGGAGGCAGAAT-3′). PCR protocol included 30 cycles with an alignment temperature of 55°C for all primer pairs used. The PCR products were run in 1% agarose gels and stained with ethidium bromide for visualization.

### Statistics

Statistical significance was tested with paired two-tailed Student's *t*-test. Data are reported as mean ± SD. A *p*-value ≤ 0.05 was considered statistically significant.

## Results

### Characterization of NETs induced by *E. histolytica* trophozoites

#### Detection of NETs components

**Figure 1:**
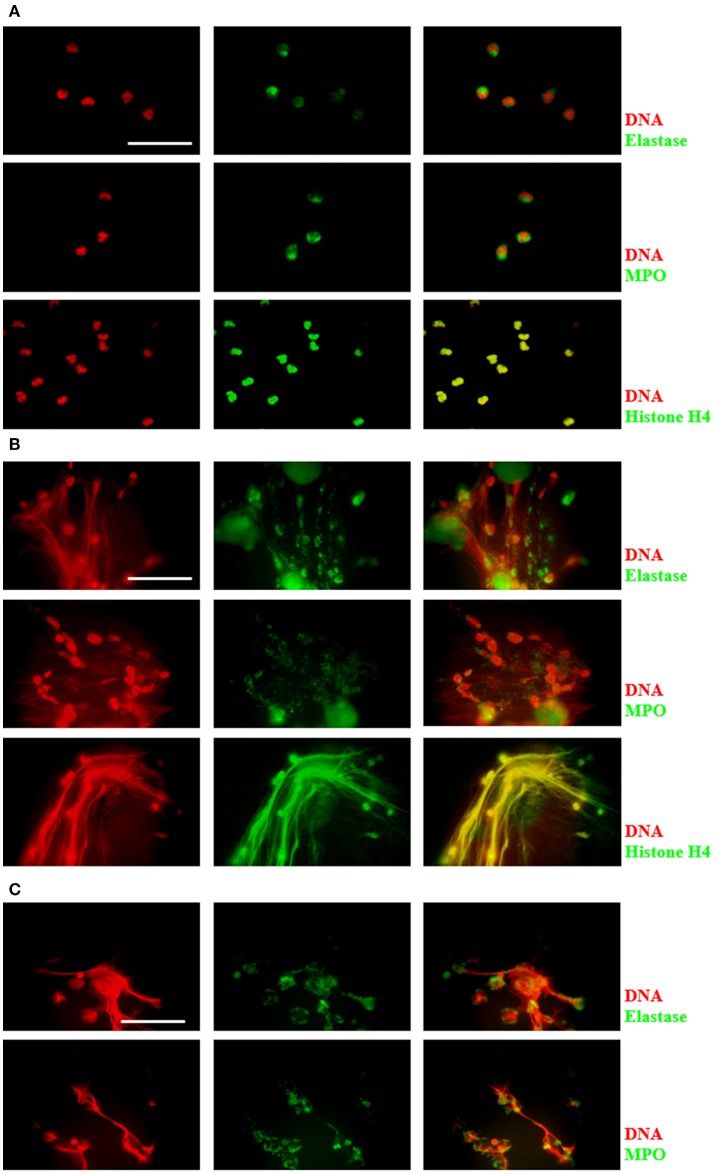
Characterization of components in NETs induced by *E. histolytica* trophozoites. Human neutrophils (2 × 10^5^) were cultured in absence of stimuli **(A)** or stimulated with 1 × 10^4^ trophozoites **(B)** or 20 nM PMA **(C)** in RPMI medium during 4 h at 37°C. Cells were fixed and immunofluorescence was performed using anti-neutrophil elastase (NE), anti-myeloperoxidase (MPO) or anti-histone H4 antibodies followed by anti-mouse IgG conjugated to FITC (for NE and MPO) or anti-rabbit IgG conjugated to TRITC (for histone H4). DNA was stained with DAPI. Images were taken at 100x magnification. Scale bar 50 μm.

DNA release during neutrophil/amoeba co-culturing has been reported previously (Ávila et al., 2016). Here, these structures were characterized to determine that they were effectively NETs. Neutrophils and amoebas were co-cultured in a proportion of 20:1, respectively, in RPMI medium supplemented with FBS 5% during 4 h. After this period, unstimulated neutrophils showed a cytoplasmic localization of NE and MPO, meanwhile, histone H4 co-localized with DNA as expected (Figure 1A). In contrast, in the neutrophil/amoeba co-culture, DNA extracellular fiber were detected by DAPI staining and these structures co-localized with proteins associated with NETs such as NE, MPO and histone H4 (Figure 1B). Similar structures were appreciated in neutrophils stimulated with 20 nM PMA, a well-characterized NETosis inductor (Figure 1C). Interestingly, while histone H4 showed a regular distribution along DNA filaments, NE and MPO were detected as immunostained spots only partially co-localized with DNA. Trophozoites were not stained with anti-NE or anti-MPO antibodies and the anti-histone H4 antibody showed a nuclear localization as expected (data not shown).

#### Scanning electron microscopy of NETs

**Figure 2:**
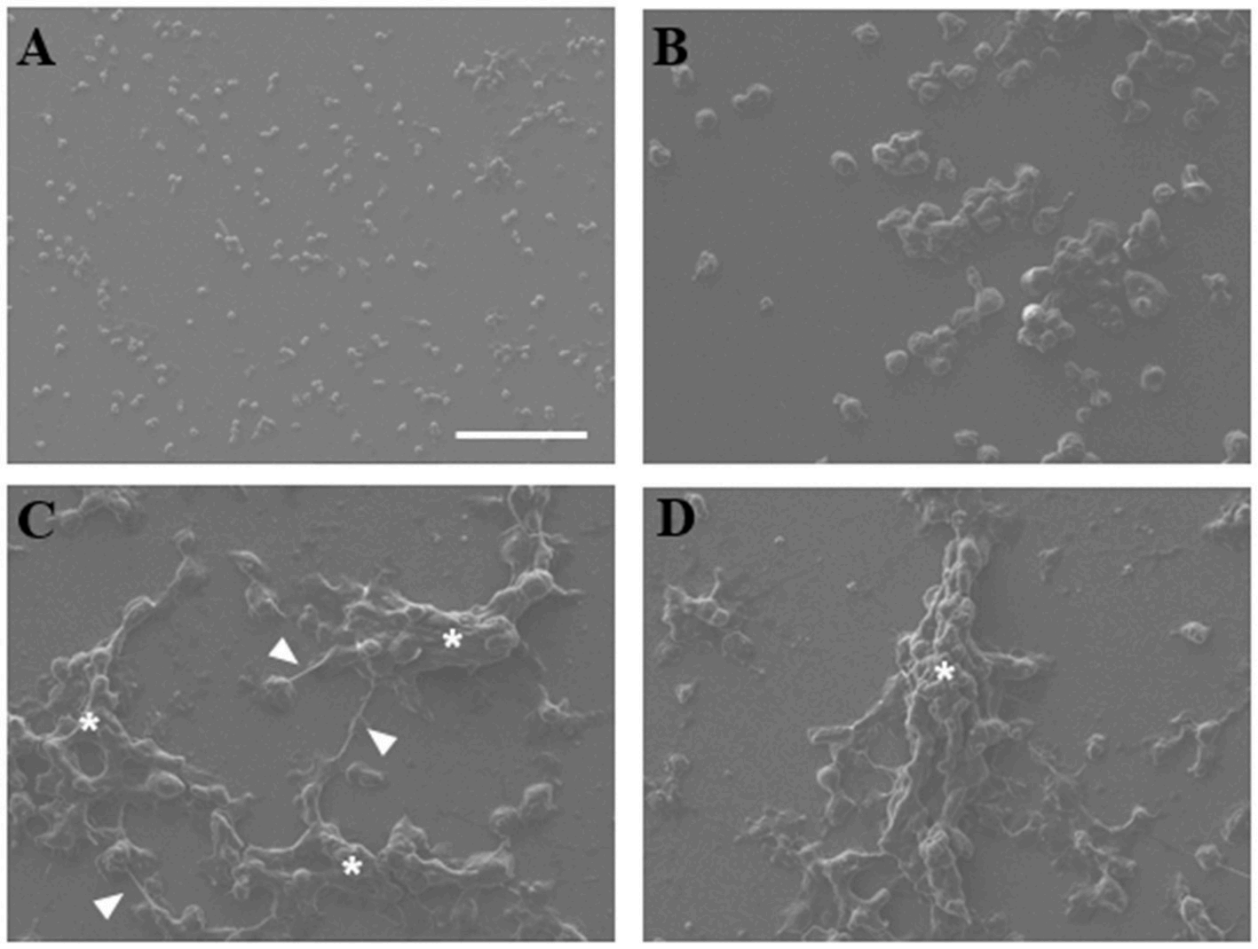
Scanning electron microscopy of NETs induced by amoebas. **(A)** Neutrophils, **(B)** trophozoites, or **(C,D)** co-cultured neutrophils-amoebas (ratio 20:1) were incubated during 4 h at 37°C. Posteriorly, cells were fixed, dehydrated, air dried, and coated with gold. Fibrillar structures (arrowhead) and clusters of amoebas trapped in the fibrillar material (asterisks) are shown. Images were taken at 250x magnification. Scale bar 100 μm.

NETs were analyzed by scanning electron microscopy to observe interaction of trophozoites with extracellular DNA. Non-stimulated neutrophils were visualized as adherent spherical cells with an irregular surface (Figure 2A), while trophozoites were detected as pleomorphic cells (Figure 2B). After neutrophil–amoeba interaction, trophozoites appeared embedded in the extracellular material (NETs) forming clusters and surrounded by fiber-like structures in close contact with amoebas (Figures 2C–D). None-intact neutrophils were visualized in this condition suggesting that all neutrophils undergo NETosis.

#### Trophozoites induce NETosis and no other forms of cell death

**Figure 3:**
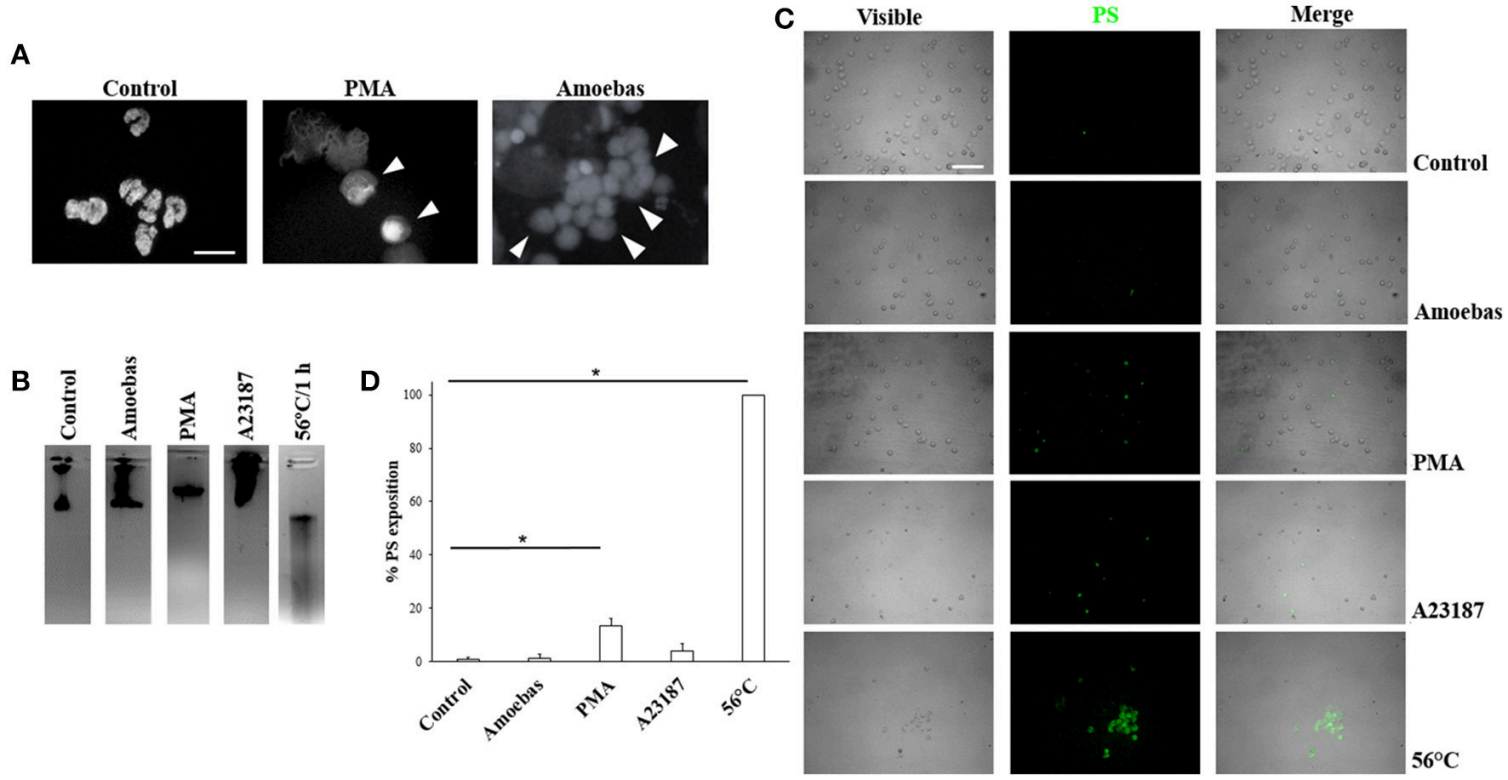
Trophozoites induces NETosis without evidence of apoptosis or necrosis. **(A)** Neutrophils were incubated with 20 nM PMA or trophozoites (ratio 20:1). After 4 h of incubation, cells were fixed and stained with DAPI. Decondensed chromatin is shown (arrowheads). Images were taken at 100x magnification. Scale bar 10 μm. **(B)** DNA from neutrophils (1 × 10^6^) treated for 1 h with trophozoites (5 × 10^4^) or 20 nM PMA or 10 μM A23187 or heat (50°C) were extracted and run in 1.8% agarose gel and the bands visualized by staining with ethidium bromide. **(C)** Phosphatidylserine (PS) exposition was assessed by fluorescence microscopy using FITC-annexin V. **(D)** Percentage of cells positive to PS was determined in a total of 300 stained neutrophils. Values are means ± SD of three independent experiments. ^*^p < 0.001.

In order to discard other forms of cell death during neutrophil–amoeba interaction, the nuclear morphology at the moments before DNA extrusion was analyzed. Non-stimulated neutrophils showed characteristic condensed-multilobular nuclei (Figure 3A), while PMA-treated neutrophils presented decondensed chromatin with loss of multilobular morphology associated to the NETosis process. During neutrophil–amoeba interaction, neutrophils also presented decondensed nuclei before DNA release suggesting NETosis process (Figure 3A). To confirm these observations, DNA from neutrophils treated with PMA, A23187, exposed to amoebas or subject to heat was run in agarose gels to verify its integrity. DNA from control neutrophils as well as neutrophils treated with PMA, A23187 or amoebas showed no fragmented DNA (Figure 3B), whereas smeared DNA was seen with heat-treated neutrophils. PMA-treated neutrophils, but above all, heat-treated neutrophils showed an increase in phosphatidylserine exposition respect to control (Figures 3C,D); meanwhile, A23187 and amoebas did not show phosphatidylserine exposition.

#### NETosis occurs rapidly and depends on the viability of amoebas

**Figure 4:**
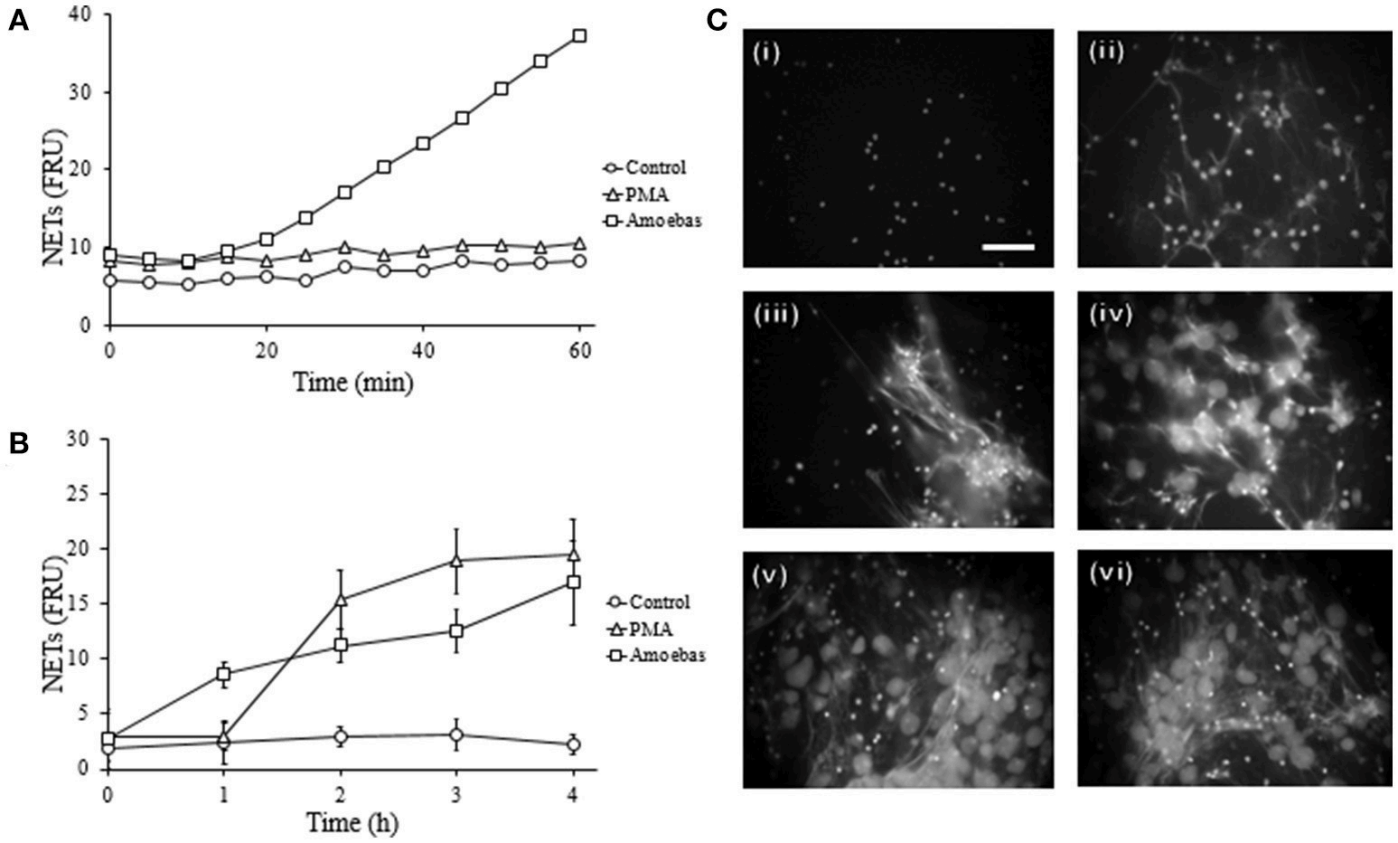
*E. histolytica* trophozoites induces a rapid NETosis process. **(A)** Neutrophils (1 × 10^5^) were co-culture with trophozoites (5 × 10^3^) in RPMI medium added with 500 nM SYTOX Green and fluorescence values were measured every 5 min during 1 h. **(B)** Neutrophils (3 × 10^5^) were co-cultured with trophozoites (1.5 × 10^4^) in RPMI medium for the indicated times; supernatants were collected, mixed 1:1 with 500 nM SYTOX Green and the fluorescence measured. In **(A,B)** 20 nM PMA was used as positive control of NETosis, NETs amount is expressed in fluorescence relative units (FRU) and values are means ± SD of three independent experiments in triplicate. **(C)** Neutrophils were stimulated with 20 nM PMA (4 h) or amoebas (ratio 20:1) during 1, 2, 3, and 4 h and the cells fixed and stained with DAPI. Control neutrophils (i), neutrophils treated with PMA (ii), neutrophils co-cultured with amoebas for 1 h (iii), 2 h (iv), 3 h (v), and 4 h (vi). Images were taken at 60x magnification. Scale bar 50 μm.

Previously, we demonstrated in a qualitative manner that *E. histolytica* trophozoites induce a rapid NETs release (Ávila et al., 2016); here, these results were verified quantitatively. We show that neutrophils extruded DNA evidently after 25 min of interaction with amoebas (Figure 4A) and the amounts of expelled DNA increased in the time-course of following 4 h (Figure 4B). PMA-treated neutrophils underwent NETosis after 1 h of stimulation and control-neutrophils did not release DNA in our experiments. These data were confirmed by fluorescence microscopy: control neutrophils were observed with multilobular and condensed nuclei (Figure 4C, panel i), PMA-treated neutrophils showed decondensed chromatin and extracellular DNA fibers (Figure 4C, panel ii), whereas in neutrophil-amoebas co-cultures, scarce intact neutrophils were observed and the trophozoites appeared surrounded by extracellular DNA fibers (Figure 4C, panels iii–vi).

**Figure 5:**
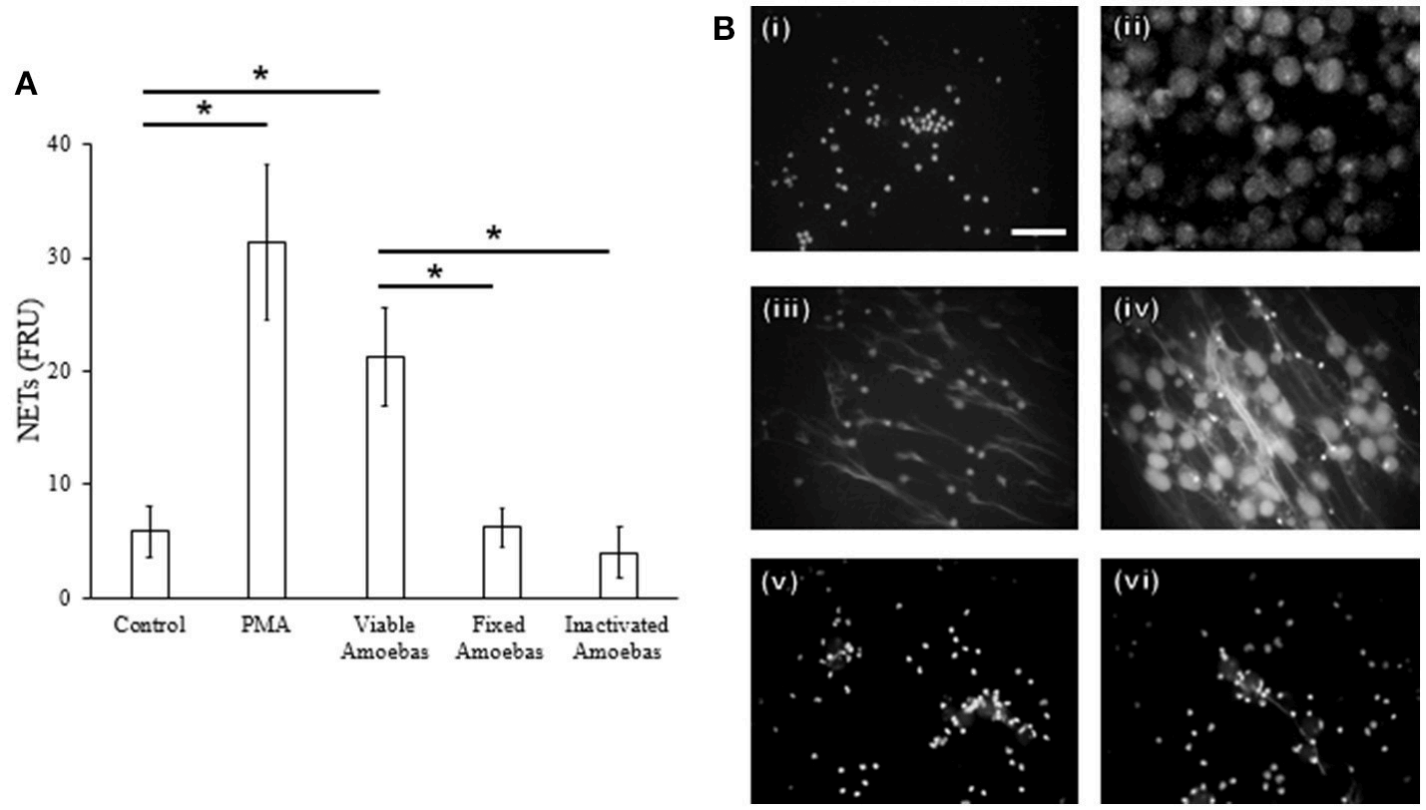
NET release depends on amoeba viability. **(A)** Neutrophils (3 × 10^5^) were co-cultured with viable, formaldehyde-fixed or heat-inactivated trophozoites (1.5 × 10^4^) in RPMI medium for 4 h. PMA at 20 nM was used as positive control of NETosis. Supernatants were collected, mixed 1:1 with SYTOX Green (500 nM) and fluorescence was measured. NETs amount is expressed in fluorescence relative units (FRU). Values are means ± SD of three independent experiments in triplicate. *p < 0.001. **(B)** Neutrophils were stimulated with 20 nM PMA or viable or non-viable amoebas (ratio 20:1) during 4 h; cells were fixed and staining with DAPI. Control neutrophils (i), control amoebas (ii), neutrophils treated with PMA (iii), neutrophils co-cultured with viable (iv), fixed (v), or heat-inactivated amoebas (vi). Images were taken at 60x magnification. Scale bar 50 μm.

Fixed amoebas have been reported to be unable to induce NETosis after 1 h of interaction with neutrophils (Ávila et al., 2016). Because some stimuli take more than 1 h to induce this phenomenon, NETs amounts were determined after 4 h of stimulation with either PMA, viable trophozoites or formaldehyde-fixed or heat-inactivated amoebas. As described before, PMA-treated neutrophils and neutrophils co-cultured with viable trophozoites extruded DNA (Figures 5A,B, panels iii and iv), but heat-inactivated or formaldehyde-fixed trophozoites failed to induce NETs after the same incubation period even when they were in close contact with neutrophils (Figures 5A,B, panels v and vi). These data suggest that the surface molecules present on the trophozoites are not the unique stimuli necessary to induce NETosis.

#### NETs contain both nuclear and mitochondrial DNA

**Figure 6:**
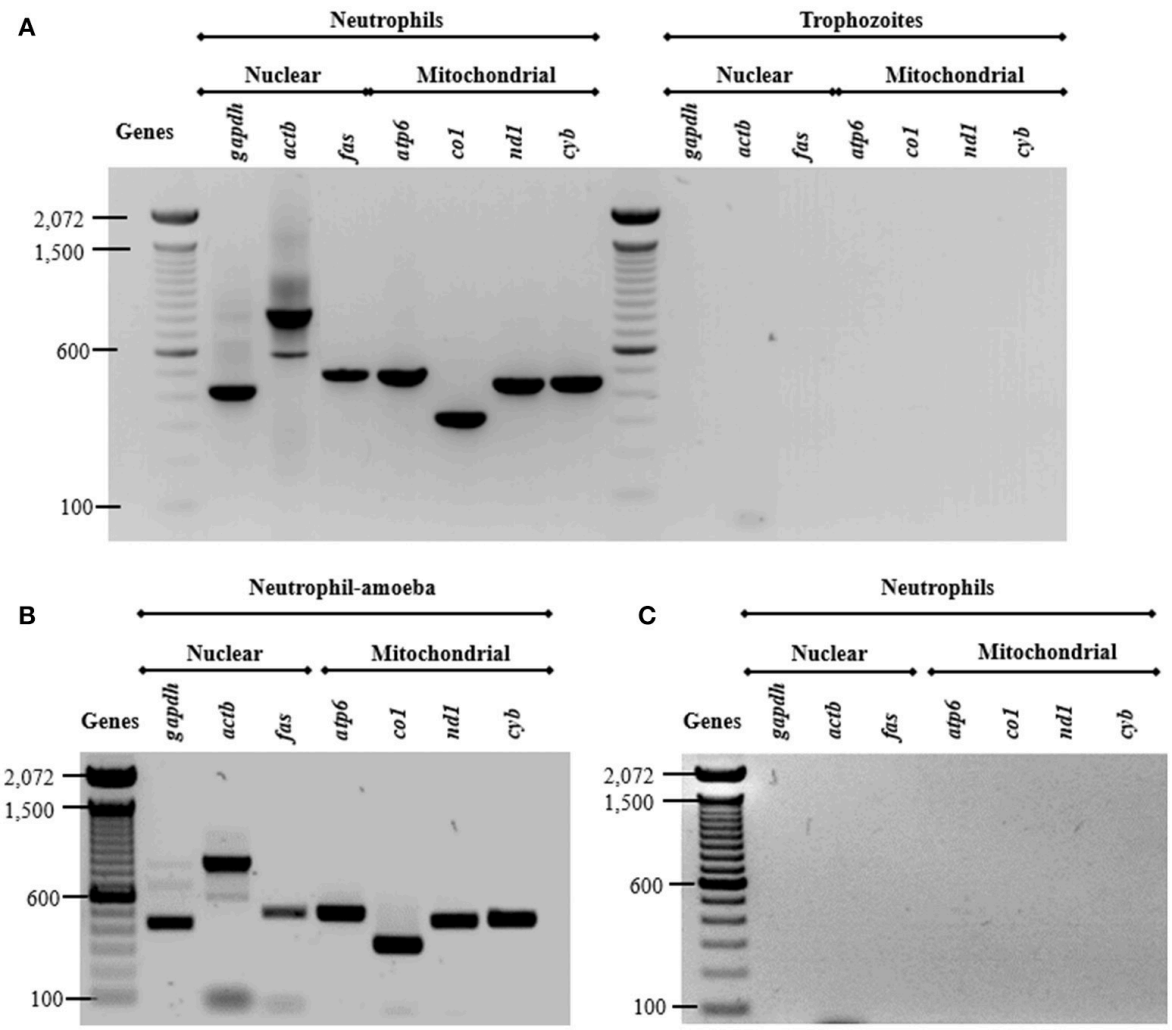
Both nuclear and mitochondrial DNA composes NETs induced by *E. histolytica* trophozoites. **(A)** DNA from neutrophils and trophozoites were extracted and PCR was performed to amplify three nuclear (*gapdh, actb*, and *fas*) and four mitochondrial (*atp6, co1, nd1*, and *cyb*) genes. Supernatant from **(B)** neutrophil (1 × 10^6^)-trophozoites (5 × 10^4^) co-cultures or **(C)** from neutrophils without any stimulus were directly used as template for PCR reactions to amplify the nuclear and mitochondrial genes. PCR amplicons were run 1% agarose gels and stained with ethidium bromide for visualization.

Previous studies show that DNA from NETs can have either nuclear or mitochondrial origin, or both, depending on stimuli used (Yousefi et al., 2009). Here, we assessed the origin of DNA from NETs amplifying three nuclear and four mitochondrial neutrophil genes. As expected, all amplicons were obtained from neutrophil DNA while no amplicons were obtained from purified *E. histolytica* DNA (Figure 6A) indicating that trophozoite DNA co-purified from NETs does not contribute to the amplification. The presence of both nuclear and mtDNA was detected from supernatant of amoebas-neutrophils co-cultured for 15 min (Figure 6B). No amplification was obtained using supernatant from PMN cultured alone for 4 h (Figure 6C).

### Dependence of NETosis process of ROS generation

#### NETs were generated independently of NOX2-derived ROS

**Figure 7:**
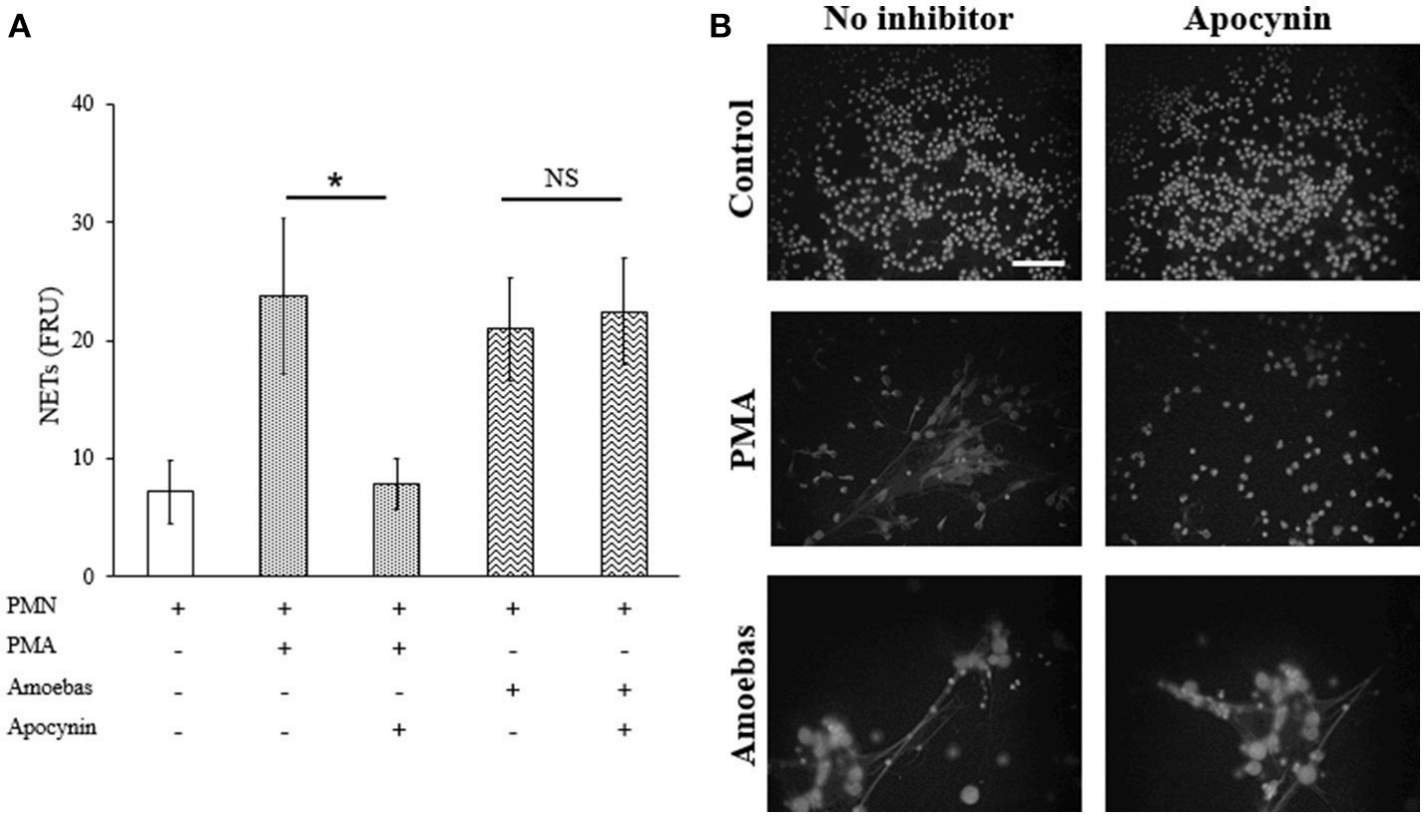
NETosis induced by trophozoites is independent of NOX2-derived ROS. **(A)** Neutrophils (3 × 10^5^) were pre-incubated with 200 μM apocynin or DMSO for 30 min and then stimulated with 20 nM PMA or co-cultured with trophozoites (1.5 × 10^4^) during 4 h. Supernatants were collected, mixed 1:1 with SYTOX Green (500 nM) and fluorescence was measured. NETs amount is expressed in fluorescence relative units (FRU). Values are means ± SD of three independent experiments in triplicate. ^*^p < 0.001; NS: statistically non-significant. **(B)** Neutrophils were treated as describe previously, cells were fixed and stained with DAPI. Images were taken at 60x magnification. Scale bar 50 μm.

To establish the mechanisms involved in the induction of NETs by amoebas, we determined the dependency of this process on NOX2-derived ROS production using apocynin as inhibitor of NOX2 activity (the widely used NOX2 inhibitor DPI caused amoebas death; data not shown). As expected, apocynin (200 μM) inhibited NETosis pretended with PMA treatment for 4 h showing that the mechanism of PMA-induced NETosis depends on ROS production. However, apocynin treatment did not decrease DNA release induced by trophozoites indicating that trophozoite-induced NETosis is independent of ROS production by NOX2 (Figure 7A). Apocynin treatment did not lead to morphological alterations in control neutrophils as well as did not affect NETs amount visualized by microscopy in neutrophils stimulated with trophozoites (Figure 7B).

#### ROS were not generated during neutrophil–amoeba interaction

**Figure 8:**
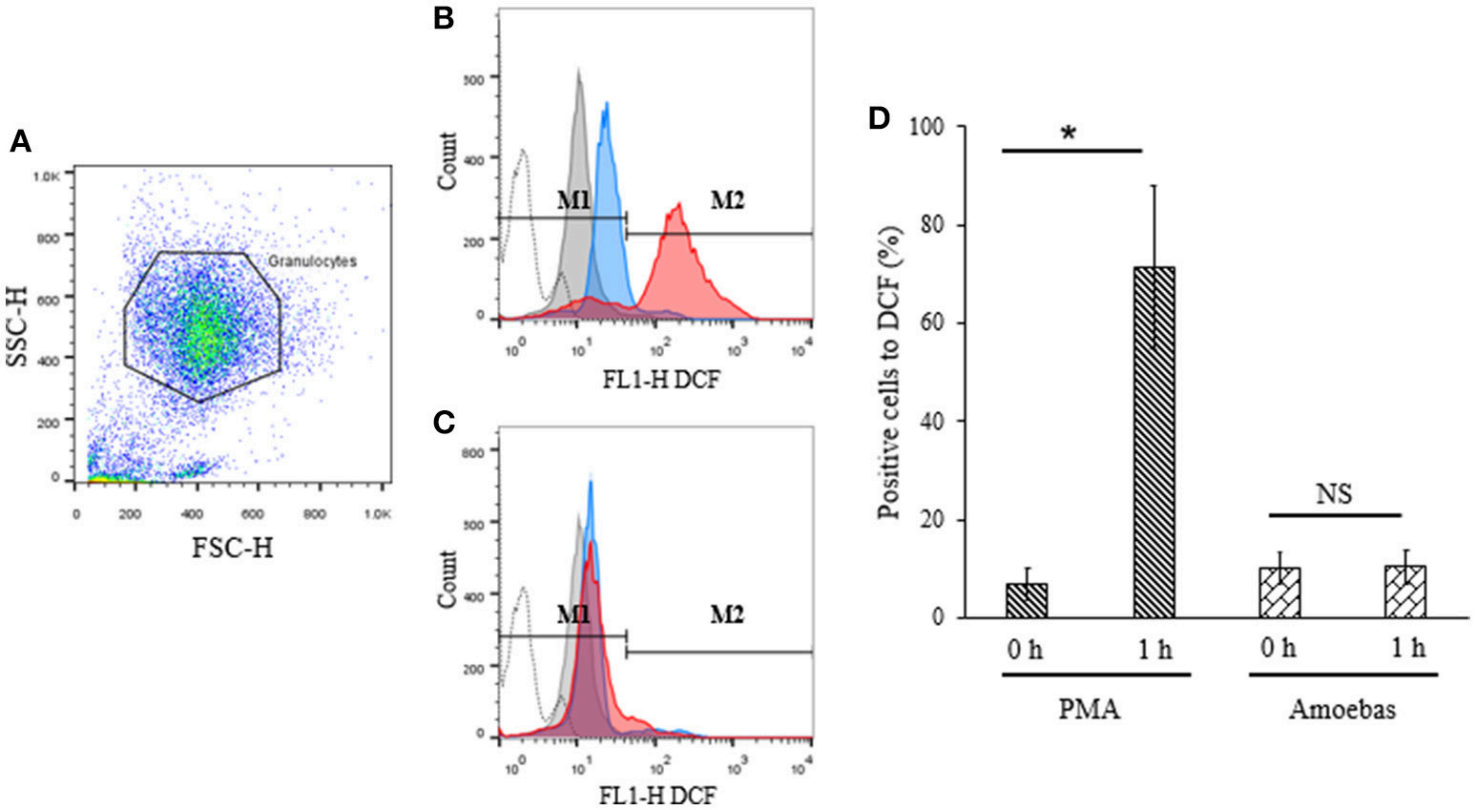
ROS were not detected during neutrophil–amoeba interaction. Neutrophils (1 × 10^6^) were pre-incubated with 10 μM H_2_DCFDA for 30 min and stimulated with 5 × 10^4^ trophozoites or 20 nM PMA for 1 h. DCF fluorescence was measured using a FACScalibur cytometer. **(A)** Neutrophils population selected for analysis. **(B)** Histogram of neutrophils stimulated with PMA. **(C)** Histogram of neutrophils co-cultured with trophozoites. M1 negative region (cells with low fluorescence); M2 positive region (cells with high fluorescence). Histograms: dotted, autofluorescence; gray, non-stimulated; blue, stimulated 0 h; red, stimulated 1 h. **(D)** Percentage of positive cells to DCF respect to the control. Values are means ± SD of three independent experiments. ^*^p < 0.001. NS, statistically non-significant.

ROS generation has been previously linked to the defense against *E. histolytica* (Denis and Chadee, 1989). For this reason, assuming that NETosis induced by this parasite was independent of NOX2-derived ROS, the production of ROS from this source was investigated during neutrophil–amoeba interaction by flow cytometry. Population corresponded to neutrophils was selected by flow cytometry according to size and granularity features (Figure 8A). Negative region M1 was defined as covering the 98% events in the absence of stimuli; positive region M2 was defined as all values superior to M1.

Basal DCF fluorescence levels were detected in PMA-stimulated neutrophils at 0 h but these increased significantly after 1 h of stimulation indicating ROS generation (Figures 8B,D). On the other hand, no significant differences were observed in neutrophils co-cultured with trophozoites after 1 h as compared to the starting time point suggesting that ROS production did not take place during interaction of neutrophils with the parasite (Figures 8C,D).

### Requirements for neutrophil elastase during NET formation

#### NE is translocated to nucleus during NETosis

**Figure 9:**
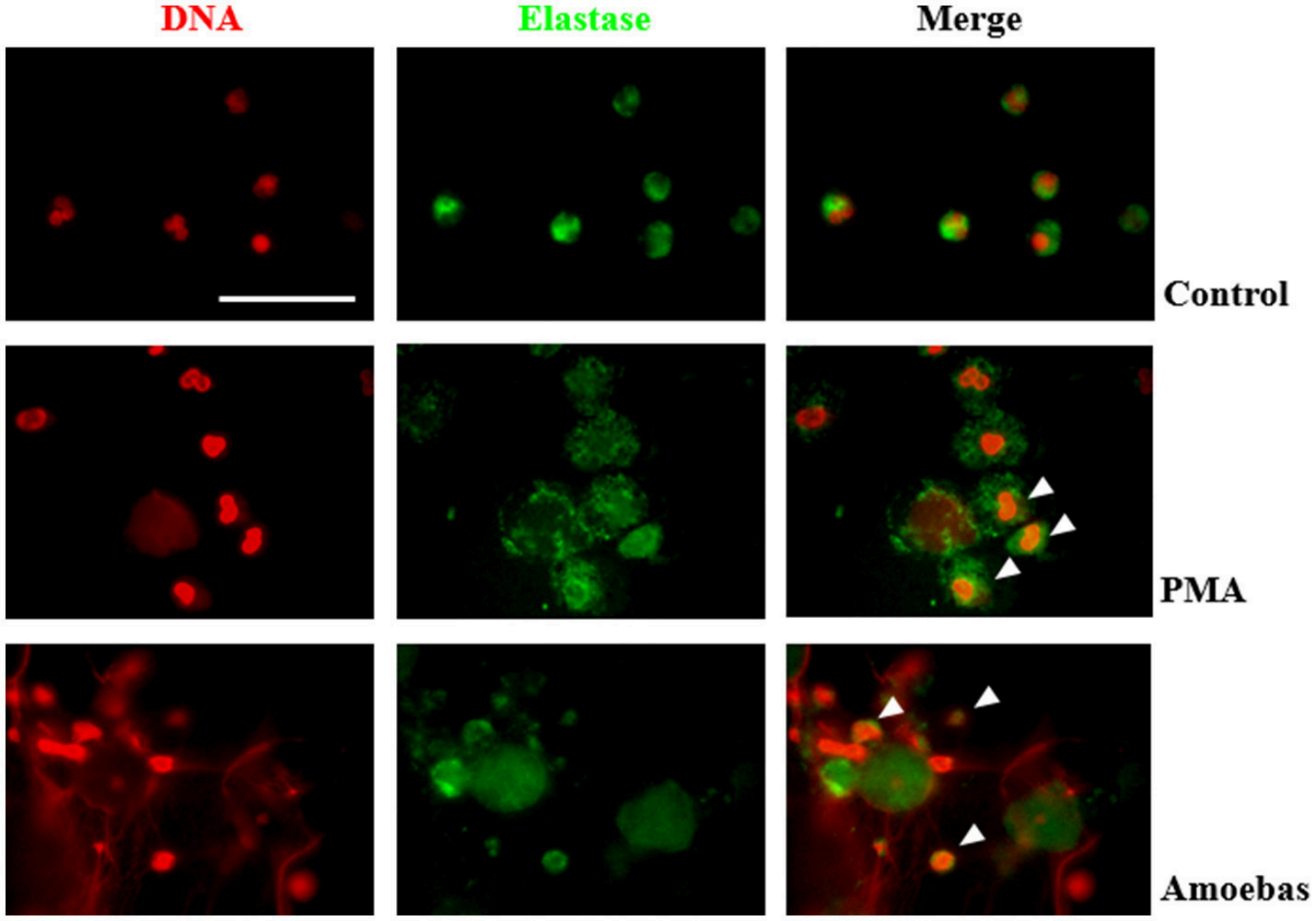
Neutrophil elastase (NE) translocation to the nucleus take place during NETosis induced by trophozoites. Non-stimulated neutrophil (2 × 10^5^) and neutrophils stimulated by 20 nM PMA or co-culture with 1 × 10^4^ trophozoites during 4 h were fixed and immunofluorescence performed using anti-NE antibody and anti-mouse IgG conjugated to FITC. DNA was staining with DAPI. Arrowheads indicate nuclear localization of NE. Images were taken at 100x magnification. Scale bar 50 μm.

Nuclear translocation of NE is a step of NETosis that has been proposed to require ROS production by NOX2 (Papayannopoulos et al., 2010). This concept was explored in our model even though amoeba-induced NETosis resulted independent of NOX2-derived ROS. By immunofluorescence, control neutrophils showed multilobular nuclei and NE was localized to cytoplasm, as expected (Figure 9), while 20 nM PMA treatment caused NE migration to nucleus after 1 h of stimulation and before DNA decondensation (Figure 9). In both cases, a NE remnant was also detected in cytoplasm. Surprisingly, neutrophils co-cultured with trophozoites also presented co-localization of the enzyme with chromatin with patterns of irregular distribution in pre-NETotic nuclei after 15 min of interaction (Figure 9). These results indicate that NE translocation to the nucleus can take place independently of ROS generation by NOX2 during NETosis induced by trophozoites.

#### Serine-protease inhibition results in reduction of NET release

**Figure 10:**
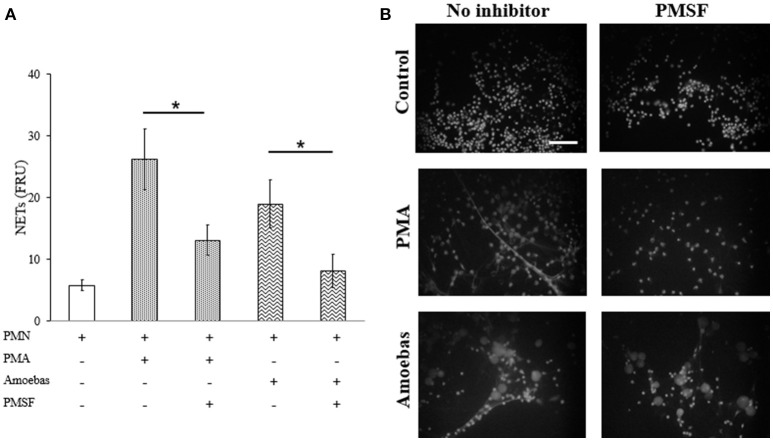
Serine protease activity is required for NETosis induce by *E. histolytica* trophozoites. **(A)** Neutrophils (3 × 10^5^) were pre-incubated with 1 mM PMSF or DMSO for 30 min and then stimulated with 20 nM PMA or co-cultured with trophozoites (1.5 × 10^4^) during 4 h. Supernatants were collected, mixed 1:1 with SYTOX Green (500 nM) and fluorescence was measured. NETs amount is expressed in fluorescence relative units (FRU). Values are means ±SD of three independent experiments in triplicate. ^*^p < 0.001. **(B)** Neutrophils were treated as describe previously, fixed and stained with DAPI. Images were taken at 60x magnification. Scale bar 50 μm.

NE is a serine-protease that decondense chromatin after its translocation to nucleus in the process of NETosis (Papayannopoulos et al., 2010). To study the role of NE in NETosis induced by *E. histolytica* trophozoites, 1 mM phenylmethylsulfonyl fluoride (PMSF) was used to inhibit its activity in neutrophils. Serine protease inhibition caused a statistically significant decrease in NET amounts released by neutrophils stimulated with 20 nM PMA or *E. histolytica* trophozoites (Figure 10A). In these experiments, PMSF did not affect cell viability (data not shown). The result was confirmed under fluorescence microscopy showing that PMSF reduced the numbers of extracellular DNA fibers in PMA- or trophozoites-treated neutrophils in respect to controls. Additionally, major numbers of non-NETotic cells were noted when neutrophils were pre-treated with PMSF (Figure 10B). These results suggest that a serine-protease, probably NE, is required to induce NET release in neutrophils co-cultured with trophozoites.

### Role of PAD4 in netosis induced by amoebas

#### Extracellular calcium is required for NETosis

**Figure 11:**
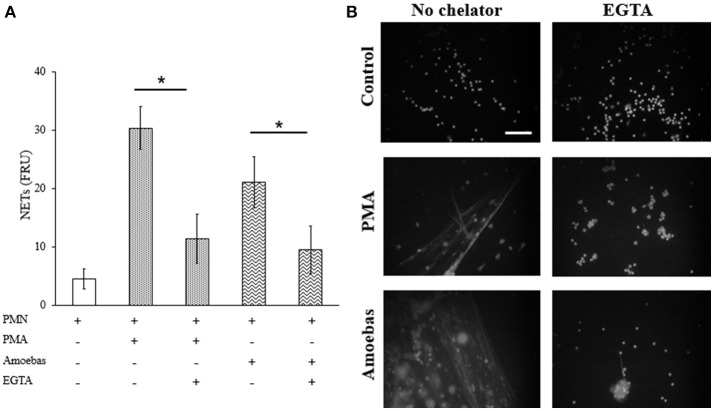
NETosis induced by *E. histolytica* trophozoites require the presence of extracellular calcium. **(A)** Neutrophils (3 × 10^5^) were culture in the presence or absence of 20 mM EGTA. EGTA-treated neutrophils were stimulated with 20 nM PMA or co-cultured with trophozoites (1.5 × 10^4^) during 4 h. Supernatants were collected, mixed 1:1 with SYTOX Green (500 nM) and fluorescence was measured. NETs amount is expressed in fluorescence relative units (FRU). Values are means ± SD of three independent experiments in triplicate. ^*^p < 0.001. **(B)** Neutrophils were treated as describe previously, fixed and stained with DAPI. Images were taken at 60x magnification. Scale bar 50 μm.

Extracellular calcium influx required for PAD4 activation was considered to be linked to NETosis processes independent of NOX2-derived ROS (Konig and Andrade, 2016). Therefore, herein calcium was chelated from the medium by adding 20 mM EGTA before neutrophil stimulation with 20 nM PMA or trophozoites. EGTA treatment did not affect cell viability of both neutrophils and amoebas (data not shown). EGTA addition reduced significantly DNA extrusion by neutrophils after stimulation with *E. histolytica* trophozoites; however, NETosis induced by PMA was also reduced (Figure 11A). This result was corroborated by fluorescence microscopy showing that extracellular DNA fibers were less abundant with respect to controls in PMA- or amoeba-treated neutrophils when calcium was previously chelated (Figure 11B). These data suggest that NETosis induced by amoeba is dependent on extracellular calcium, which appears to be required for NETosis process independently of requirements for PAD4 activity as PMA-induced NETosis do not depend on PAD4.

#### PAD4 inhibition do not affect NET release

**Figure 12:**
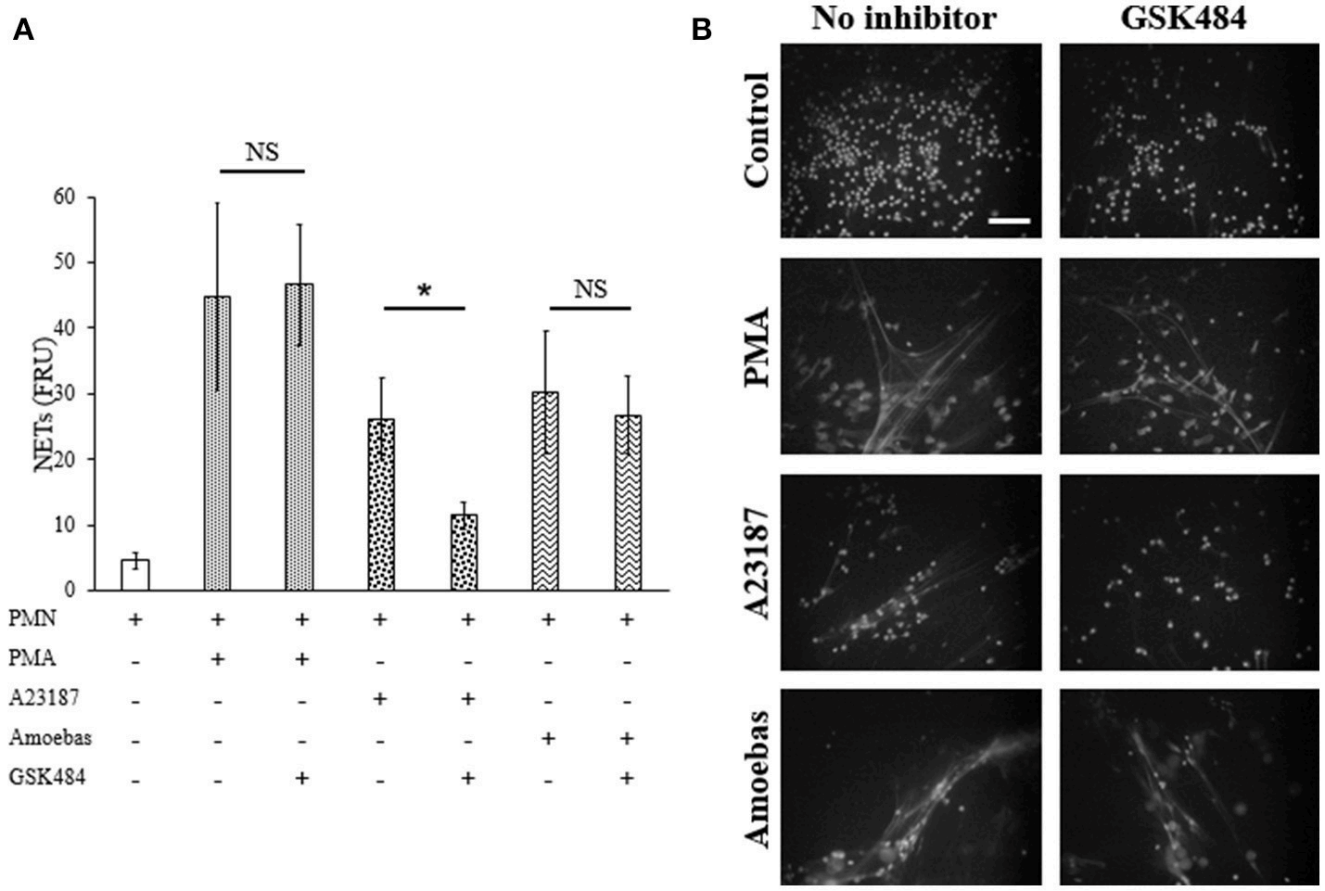
NETosis induced by *E. histolytica* trophozoites is independent of PAD4 activity. **(A)** Neutrophils (3 × 10^5^) were pre-incubated with 10 μM GSK484 or DMSO for 30 min and then stimulated with 20 nM PMA, 10 μM A23187, or co-cultured with trophozoites (1.5 × 10^4^) during 4 h. Supernatants were collected, mixed 1:1 with SYTOX Green (500 nM) and fluorescence was measured. NETs amount is expressed in fluorescence relative units (FRU). Values are means ±SD of three independent experiments in triplicate. ^*^p < 0.001. NS: statistically non-significant. **(B)** Neutrophils were treated as describe previously, fixed and stained with DAPI. Images were taken at 60x magnification. Scale bar 50 μm.

Extracellular calcium dependent-NETosis has been associated to DNA decondensation by PAD4 activity (Douda et al., 2015). In order to explore the participation of PAD4 in NETosis induced by *E. histolytica* trophozoites, GSK484 was used to inhibit its activity. Neutrophils treated with 10 μM GSK484 were able to form NETs after stimulation with PMA; on the other hand, neutrophils stimulated with the calcium ionophore A23187, a known inducer of PAD4-dependent NETosis, decreased NET release when neutrophils were pre-treated with GSK484 (Figure 12A). Surprisingly, neutrophils pre-treated with PAD4 inhibitor and co-cultured with amoebas retained their capacity to form NETs (Figure 12A). As seen by fluorescence microscopy, GSK484 did not affect the morphology of neutrophils in respect to controls, and no differences were detected in the release of NETs between neutrophils treated or not with the inhibitor after stimulation with PMA (Figure 12B). In a similar way, PAD4 inhibition did not affect NET release in neutrophils co-cultured with trophozoites; however, neutrophils pre-treated with GSK484 formed less NETs upon stimulation with A23187 (Figure 12B).

#### Detection of citrullinated proteins

**Figure 13:**
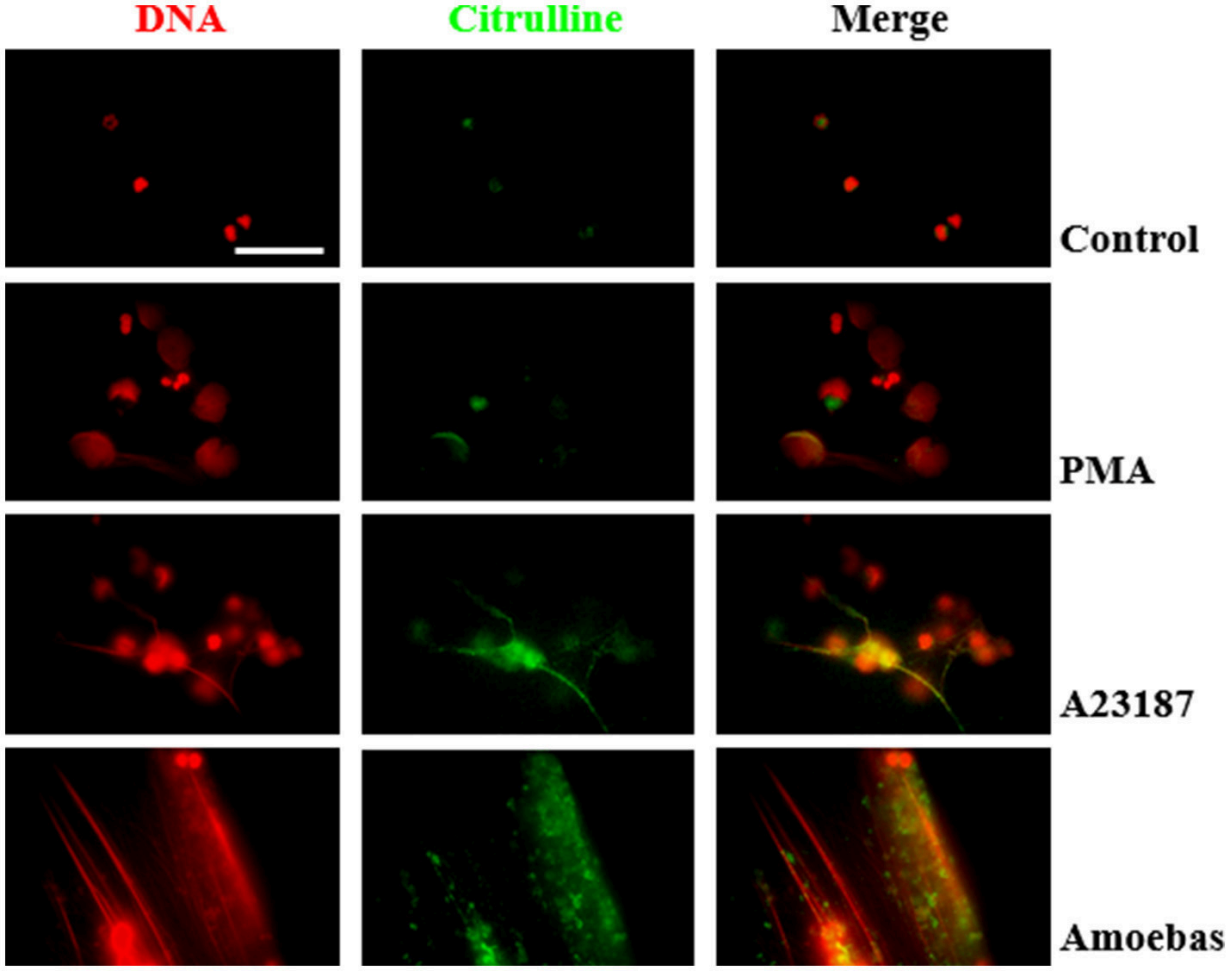
Protein citrullination occurs during NET release induce by *E. histolytica* trophozoites. Non-stimulated neutrophil (2 × 10^5^) and neutrophils stimulated with 20 nM PMA, **(C)** 10 μM A23187 or co-cultured with 1 × 10^4^ trophozoites during 4 h were fixed and immunofluorescence performed using anti-citrulline antibody and anti-rabbit IgG conjugated to TRITC. DNA was staining with DAPI. Images were taken at 100x magnification. Scale bar 50 μm.

Citrullinated proteins are considered as markers of PAD4-mediated NETosis (Wang et al., 2009). Here we assayed if NETosis induced by trophozoites generated citrullination of neutrophil proteins even though PAD4 activity was not required for NETosis process. Control and PMA-treated neutrophils showed few citrullinated proteins, principally in cytoplasm, and were less frequent in the decondensed chromatin of PMA-induced neutrophils (Figure 13). Conversely, citrullinated proteins were abundant in NETs induced by A23187 or *E. histolytica* trophozoites, co-localizing with extruded DNA in both cases. Interestingly, citrullinated proteins were detected in a spot pattern similar to the observed during the detection of NE in NETs induced by trophozoites. These results suggest that protein citrullination take place in the NETosis triggered by amoeba.

## Discussion

NETosis is a relatively new mechanism of innate immunity that has been linked to the defense against different pathogens including bacteria, fungi and protozoa by killing or inhibiting their growth, preventing their spread and contributing to the establishment of a protective immune response against pathogens (Brinkmann et al., 2004; Urban et al., 2006; Guimarães-Costa et al., 2009; Röhm et al., 2014; Halverson et al., 2015; Sousa-Rocha et al., 2015). However, contradictory reports have emerged since the formation of NET seems to depend on the pathogen size, with the large pathogens or aggregates of the small ones being responsible for triggering NETosis, while the small non-aggregated pathogens are targeted by phagocytosis (Branzk et al., 2014). In addition, several microorganisms evade the action of NETs by inhibiting their release, degrading the DNA with nucleases or resisting the anti-microbial effect of the NET-associated proteins through encapsulation (reviewed in Storisteanu et al., 2017). On the other hand, the excessive development of NETs has recently begun to be associated with autoimmune and vasculitic diseases, contributing in general to the pathology of some diseases associated with microbial infections (Yipp and Kubes, 2013). Therefore, the role of NETs in the outcome of most infectious diseases is still unknown and is a matter of intensive studies. Nevertheless, the mechanisms underlying the formation of NETs remain poorly understood.

*E. histolytica* is a parasite that causes intestinal amoebiasis and, in some cases, amoebic liver abscesses. Very early in both pathologies, neutrophils are rapidly recruited to the site of infection becoming the cell of the innate immunity more prevalent in the lesions. However, the role of neutrophils in the amoebic infection is still not well understood. Studies on the outcome of the interaction of neutrophils with amoeba *in vitro* have shown that priming of these cells with recombinant cytokines IFN-γ and TNF-α make them capable to kill 97% of *E. histolytica* trophozoites in a H_2_O_2_-dependent manner (Denis and Chadee, 1989). In contrast, non-primed neutrophils suffer apoptosis and/or are highly phagocyted when exposed to virulent *E. histolytica* trophozoites (Sim et al., 2004; Ávila et al., 2016). Likewise, *in vivo*, while some reports confer to neutrophils a protective activity during the infection (Asgharpour et al., 2005), evidence exists that immune cells, including neutrophils, are implicated in tissue damage (Olivos-García et al., 2007). More recently, we have reported that *E. histolytica* trophozoites were capable of inducing NETosis in human neutrophils (Ávila et al., 2016), but the role of this process in amoebiasis and the molecular mechanisms implicated in its formation were not clarified. Herein we study some important characteristics associated to the NETosis process induced by neutrophil–trophozoite interaction.

First, to verify the NETosis occurrence in neutrophils co-cultures with amoebas, the presence of NETs components was tested. Like other reports, NE, MPO and histone H4 (Brinkmann et al., 2004; Kaplan and Radic, 2012) were found co-localized with extracellular DNA released by neutrophils after their interaction with trophozoites. By scanning electron microscopy, NETs were observed as extracellular fibers trapping clusters of amoebas in a similar way observed with other pathogens (Guimarães-Costa et al., 2009; Brinkmann and Zychlinsky, 2012; Della Coletta et al., 2015). Afterwards, we determined that during neutrophil–amoeba interaction the NETosis process took place and no other forms of cell death. As expected, the NETosis inducer PMA caused nuclear decondensation on neutrophils without affecting DNA integrity; however, an increase in the exposition of phosphatidylserine occurred in < 20% of the treated neutrophils, which agrees with a report showing that PMA induces phosphatidylserine exposition in neutrophils (Saito et al., 2005). Necrotic and at lesser extent apoptotic neutrophils induced by treatment with heat showed DNA degradation and phosphatidylserine exposition as it has been reported in other cells (Krysko et al., 2004; Li and Zhou, 2016). Different reports indicate that *E. histolytica* trophozoites induce apoptosis in diverse cell types including neutrophils (Seydel and Stanley, 1998; Huston et al., 2000; Sim et al., 2004). However, we show that under our conditions, the interaction of trophozoites with human neutrophils resulted in rapid decondensation of the neutrophil nuclei without phosphatidylserine exposition in their external surfaces or genomic DNA breakdown, followed by DNA release to the extracellular space, indicating that neutrophils undergo NETosis and discarding both apoptosis and necrosis in the presence of amoebas. The result was similar to the effect obtained with the calcium ionophore A23187, another NETosis inductor, which did not affect the integrity of the neutrophils DNA and prevented phosphatidylserine exposition in the external surface. Together, the results suggest at this point that amoebas and the calcium ionophore could share similar NETosis pathways which are different to those of PMA.

The most important component of NETs is the DNA released from the neutrophils. Early studies of NETosis showed that traps were exclusively generated from nuclear DNA (nDNA) (Fuchs et al., 2007). Posteriorly, it was observed that under specific conditions, neutrophils were able to form NETs exclusively from mtDNA without compromise the cell viability (Yousefi et al., 2009). Here, we showed that NETs triggered by *E. histolytica* trophozoites contain both nDNA and mtDNA, as it has been reported using other stimuli as PMA and the nitric oxide donor DETA-NONOate (Keshari et al., 2012). The release of mtDNA during NETosis has been linked to autoimmune pathologies such as eritematous systemic lupus due to its pro-inflammatory properties (Lood et al., 2016). Thus, mtDNA of NETs could cause a positive feedback through TLR9 propitiating that more neutrophils enter to NETosis (Itagaki et al., 2015). In this regard, we can speculate that mtDNA of NETs induced by trophozoites could contribute to the pathogenesis of the disease promoting inflammation, a well-known feature of the tissular damage caused by *E. histolytica*. However, we cannot rule out that mtDNA could come from neutrophils lysed during the interaction with amoebas. Therefore, further studies using specific mtDNA markers are necessary to confirm that NETs induced by amoebas effectively contain this type of DNA.

Initially, NETosis process was described as a slow mechanism when compared with phagocytosis and degranulation (Fuchs et al., 2007), taking ~2 h since addition of stimulus until DNA release. In contrast, *E. histolytica* trophozoites induced NET release on few minutes after the contact with neutrophils, similarly to other stimuli as ionomicyn, *Staphylococcus aureus* or *Leishmania amanzonensis* promastigotes (Pilsczek et al., 2010; Douda et al., 2015; Rochael et al., 2015). It is worth to mention that these rapid stimuli were shown to induce NETosis through a distinct mechanism compared to PMA and recent evidence effectively suggest that the NETosis process varies depending on the stimuli used (Muth et al., 2017). In this regard, the mechanism underlying the formation of NETs induced by amoebas seems to be non-classical. Thus, ROS production was not detected when human neutrophils were co-cultured with *E. histolytica* trophozoites in our study and apocynin, a NOX2 inhibitor, failed to inhibit amoeba-induced NETosis. Since generation of ROS has been reported during the interaction of neutrophils with *E. histolytica* trophozoites at higher ratio of trophozoites that the ratio we used here (10:1 vs. 20:1; Sim et al., 2004, 2005), our results suggest that the mechanism of NETosis induced by amoebas is independent of high concentration of ROS, or at least, of ROS generated from NOX2. Like amoebas, different stimuli induce NETosis independently of NOX2 activity including calcium ionophores, uric acid or immune complexes (Parker et al., 2012; Arai et al., 2014; Kraaij et al., 2016). Nevertheless, we detected that amoebas induced NE translocation to nucleus of neutrophils, an important step during NOX2-dependent NETosis that requires ROS formation (Papayannopoulos et al., 2010; Metzler et al., 2014). Since ROS-independent NE translocation has not been described and evidence suggests that histone cleavage takes place in NOX2-independent NETosis (Muth et al., 2017), we speculate that NOX2-independent ROS could be participating in the NETosis triggered by amoebas promoting NE translocation to the nucleus. Recently, other sources of ROS such as the mitochondrial respiratory chain or exogenous hydrogen peroxide produced by microorganisms have been considered necessary for NETosis induced by calcium ionophores and *Candida albicans* hypha, respectively (Douda et al., 2015; Muth et al., 2017). Therefore, we cannot discard that another ROS source, producing amounts that might not be detected with the methodology we used here, play a crucial role in the amoeba-induced NETosis. On the other hand, the translocation of NE to the nucleus in combination with our result showing that the inhibition of its serine protease activity with PMSF reduced the amount of NETs induced by amoeba, confirms that NE translocation is an important step in this mechanism.

PAD4 is a peptidyl arginine deiminase that catalyzes the conversion of arginine into citrulline residues in histones and requires calcium for activation (Jones et al., 2009; Bicker and Thompson, 2013). PAD4 activity is relevant to decondense DNA in some NETosis processes independent of NOX2-derived ROS and in the NETosis induced by the non-regulated calcium influx caused by calcium ionophores or calcium channel-forming proteins (Wang et al., 2009; Li et al., 2010; Konig and Andrade, 2016). Since *E. histolytica* possess amoebapores (Lynch et al., 1982), pore-forming peptides implicated in pathogenicity (Bracha et al., 2002; Zhang et al., 2004), we tested in our model the role of PAD4 in amoebic NETosis. Noteworthy, PAD4 inhibition did not affect amoebic NETosis but chelation of extracellular calcium does. This controversy can be explained by the fact that calcium is required for neutrophil adhesion, which in turn, is important for NETosis since neutrophils in suspension produce less NETs compared to adherent neutrophils (Fuchs et al., 2007). Is worth to mention that in contrast to amoebas, PAD4 inhibition reduced NETosis induced by calcium ionophore A23187, suggesting that at this point of nuclear decondensation, the NETosis processes triggered by amoebas and A23187 are different.

Because of its activity, the presence of citrullinated proteins, principally histones, is considered as a marker of PAD4-mediated NETosis (Brinkmann et al., 2016; Li et al., 2017). In accordance to other reports, our results showed that citrullinated proteins were scarce in control and PMA-treated neutrophils (Neeli and Radic, 2013; Konig and Andrade, 2016; Muth et al., 2017); however, abundant citrullinated proteins were seen in neutrophils treated with A23187 and amoebas. Considering that inhibition of PAD4 using GSK484 failed to inhibit NET release induced by amoebas, the results together suggest that protein citrullination take place but it could be no necessary in the NETosis induced by trophozoites. Further experiments are being carried out to elucidate the participation of citrullination in the process. In this regard, it was recently suggested that protein citrullination can be a mechanism that microorganisms employ to evade the immune response by inactivating the antimicrobial action of NETs proteins (Konig and Andrade, 2016). This notion may explain our previous observation that NETs failed to kill trophozoites *in vitro* and did not ever reduced their capacity to cause amoebic liver abscesses in hamsters (Ávila et al., 2016).

In conclusion, our results show that *E. histolytica* trophozoites trigger NETosis in human neutrophils by a non-classical pathway independent of NOX2-derived ROS and PAD4 activity but dependent of extracellular calcium and the serine protease activity of NE translocated to the nucleus. Our data contribute to understand how NETs are formed in the presence of *E. histolytica* trophozoites. However, more experiments are required to completely elucidate the mechanism of NETs formation triggered by this parasite and its role in protection or pathogenesis of amoebiasis.

## Author contributions

JC and CD-G conceived and designed the experiments. CD-G, ZF, and MN performed the experiments. CD-G, JC, and CR analyzed the data. JC, JL, and CR contributed reagents, materials, and analysis tools. JC and CD-G wrote the paper.

### Conflict of interest statement

The authors declare that the research was conducted in the absence of any commercial or financial relationships that could be construed as a potential conflict of interest.
